# Laparoscopic versus open lumbar sympathectomy in critical limb threatening ischemia patients in Egypt

**DOI:** 10.1186/s12893-024-02618-6

**Published:** 2024-11-07

**Authors:** Wael E. Shaalan, Ali A. Elemam, Hassan Lotfy, Ahmad R. Naga, Mohamed I. Mohamed, Yomna E. Dean, Tamer N. Abdelbaki

**Affiliations:** 1 Vascular Surgery Department, Alexandria University, Faculty of Medicine, Alexandria, Egypt; 2 Alexandria University, Faculty of Medicine, Alexandria, Egypt; 3 General Surgery Department, Alexandria University, Faculty of Medicine, Alexandria, Egypt

**Keywords:** Sympathectomy, Laparoscopic surgery, Chronic limb-threatening ischemia, Laparoscopic sympathectomy, Lumbar sympathectomy, Sympathetic denervation, Laparoscopy, Peritoneoscopy, Laparoscopic assisted surgery, Critical limb ischemia, Peripheral artery disease, Peripheral arterial disease

## Abstract

**Purpose:**

The treatment of critical limb-threatening ischemia (CLTI) is revascularization. Lumbar sympathectomy (LS) could be attempted when this is not amenable. Using laparoscopic techniques to perform LS adds the advantages of minimally invasive surgery.

**Methods:**

Twenty-four patients, presenting with non-reconstructable CLTI and rest pain, were randomly divided into group I (14 patients) who underwent retroperitoneoscopic lumbar sympathectomy (RPLS) and group II (10 patients) who had conventional open lumber sympathectomy (COLS).

**Results:**

RPLS patients had shorter hospital stays, fewer intraoperative complications, and less postoperative pain. However, the mean operative time was significantly longer (86.4 ± 9.1 min, *p*-value: 0.02) in the RPLS group but decreased with each subsequent case after that. The differences in post-operative capillary refill time, ABI, TBI, and TcPO2 were not statistically significant between both groups (*p*-values: 0.97, 0.13, 0.32, 0.10, respectively). However, the difference in the quality-of-life score was statistically significant; the mean (± SD) SF-36 score increased from 48 ± 6.8 to 81 ± 4.4 (*p*-value < 0.001) in RPLS group compared to 52 ± 8.8 to 59 ± 1.2 (*p*-value: 0.52) in COLS group.

**Conclusion:**

RPLS is feasible, safe, and has the advantages of minimally invasive surgery: minimal blood loss, less intraoperative complications, shorter hospital stay, and less postoperative pain. However, the operative time in RPLS cases is longer than in the COLS; training on the procedure is recommended to improve the learning curve.

**Supplementary Information:**

The online version contains supplementary material available at 10.1186/s12893-024-02618-6.

## Introduction

Critical limb-threatening ischemia (CLTI) is a clinical syndrome defined by the presence of peripheral artery disease (PAD), resulting in rest pain, gangrene, or lower limb ulceration lasting for more than two weeks accompanied by ankle pressure < 70 mmHg or toe pressure < 50 mmHg. CLTI is associated with increased incidence of amputation, mortality, and impaired quality of life [1]. Arterial reconstruction remains the gold standard for improving arterial perfusion, controlling symptoms, promoting ulcer healing, and preventing amputation [2] [3]. Nevertheless, when revascularization is not feasible, and there is no excessive foot necrosis, the management of rest pain is a challenge, and many patients may require amputation just for pain relief [4, 5]; hence, necessitating the use of lumbar sympathectomy (LS) [6–8].

The lumbar sympathetic chain of ganglia supplying the legs is usually located at the levels of L2 through L4, lying at the medial margin of the psoas muscle. The aorta lies anterior and medial to the left lumbar sympathetic chain, while the inferior vena cava is anterior to the right lumbar sympathetic chain [9]. Disrupting the sympathetic ganglia at the levels of L2-L4 leads to decreased vasomotor tone and decreased afferent pain signals, providing symptomatic relief.

Two methods have been described for surgical sympathectomy procedures: the conventional open LS (COLS) and retroperitoneoscopic LS (RPLS). The conventional open LS procedure carried through a retro-peritoneal approach is associated with significant disadvantages, such as the need to use muscle cutting-splitting incision, which leads to substantial postoperative complications such as pain, incisional hernia, prolonged recovery, and work leave [10]. The use of the retro-peritoneoscopic technique, however, combines the advantages of minimally invasive surgery (less postoperative pain, short hospital stays, fast recovery and return to work) and the reliability of an established open procedure [2, 11–14].

The present work aims to carry out a prospective study to compare the outcome of RPLS to the COLS in managing unreconstructable CLTI.

## Patients and methods

This is an interventional study on patients presenting with non-reconstructable CLTI admitted to the vascular surgery department between February and July 2021. Patients were randomly divided into two groups using a closed envelope method: group I: RPLS and group II: COLS. Simple randomization was performed using computer-generated numbers. To allocate each patient to their respective group, we used sequentially numbered and opaque envelopes that were sealed and only opened once the patient agreed to being part of the trial. Blinding of patients was deemed infeasible due to the obvious differences in incision sites and sizes between the two surgical techniques. Non-reconstructable CTLI was defined as Fontaine Classification III or IV with rest pain and trophic changes, an ankle-brachial index (ABI) value less than 0.5, an ankle pressure less than 50, and a toe pressure less than 30. We deemed patients ineligible for revascularization and indicated for LS in a MDT meeting including a diabetologist/foot surgeon, an anesthetist, a cardiologist, and a vascular surgeon. These patients had no outflow artery that could allow uninterrupted direct flow to the foot, no suitable conduit for bypass, or advanced medical comorbidities for which revascularization would risk the patient’s life. The study excluded any patient with extensive tissue loss necessitating amputation or those with previous major abdominal surgery. On admission, the demographic data, incidence and duration of the presenting symptoms, and clinical and vascular examination results were recorded. These included examination of peripheral pulses, capillary refill time, ABI, TBI (Toe-brachial index) [15], and transcutaneous oxygen (TcPO2) measurement [16]. A visual analog scale (VAS) assessed rest pain. Primary endpoints were the intraoperative findings, the duration of the procedure, blood loss, the ability to localize the lumbar sympathetic chain (technical success), operative injuries, and length of hospital stay. Secondary endpoints were recorded during the patient’s monthly follow-up at the outpatient vascular clinic for three months postoperatively. Patients were assessed as regards clinical and microcirculatory assessment, relief of rest pain based on the visual analog scale (VAS), ulcer healing, need for amputation, and any postoperative complications (retroperitoneal bleeding, wound pain, wound bleeding, infection, dehiscence, hernia). Moreover, a quality-of-life assessment was conducted using the 36-item Short Form Health Survey (SF-36) [17]. To prevent detection bias, all outcome assessors of secondary endpoints during the monthly follow-up visits were blinded to the intervention technique.

### Operative technique

The positioning of the patients was in a supine lateral position with a slight elevation of the flank by a bridge of the operative table Figure 2.

### Conventional open technique: COLS

A transverse oblique incision starting at the lateral edge of the rectus muscle and ending at the anterior axillary line between the anterior superior iliac spine and last rib is carried out. The external oblique, internal oblique, and the transversalis muscles are split in the direction of their fibers. The lateral plane between the transversalis fascia and the peritoneum is easily developed by blunt finger dissection toward the vertebral column. Tactile and visual identification of the lumbar sympathetic chain, located medial to the psoas muscle, was performed.

### Laparoscopic technique: RPLS (Fig. 1)

**Fig. 1:**
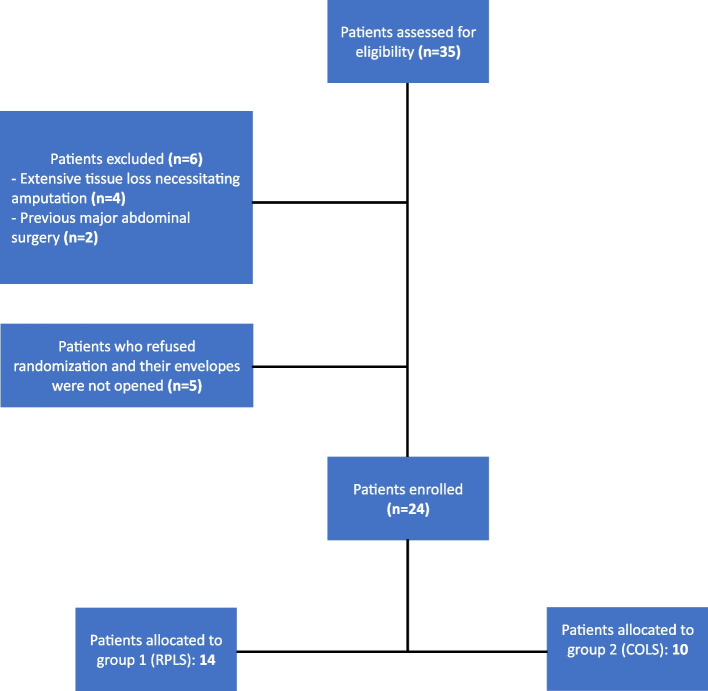
Flowchart

**Fig. 2:**
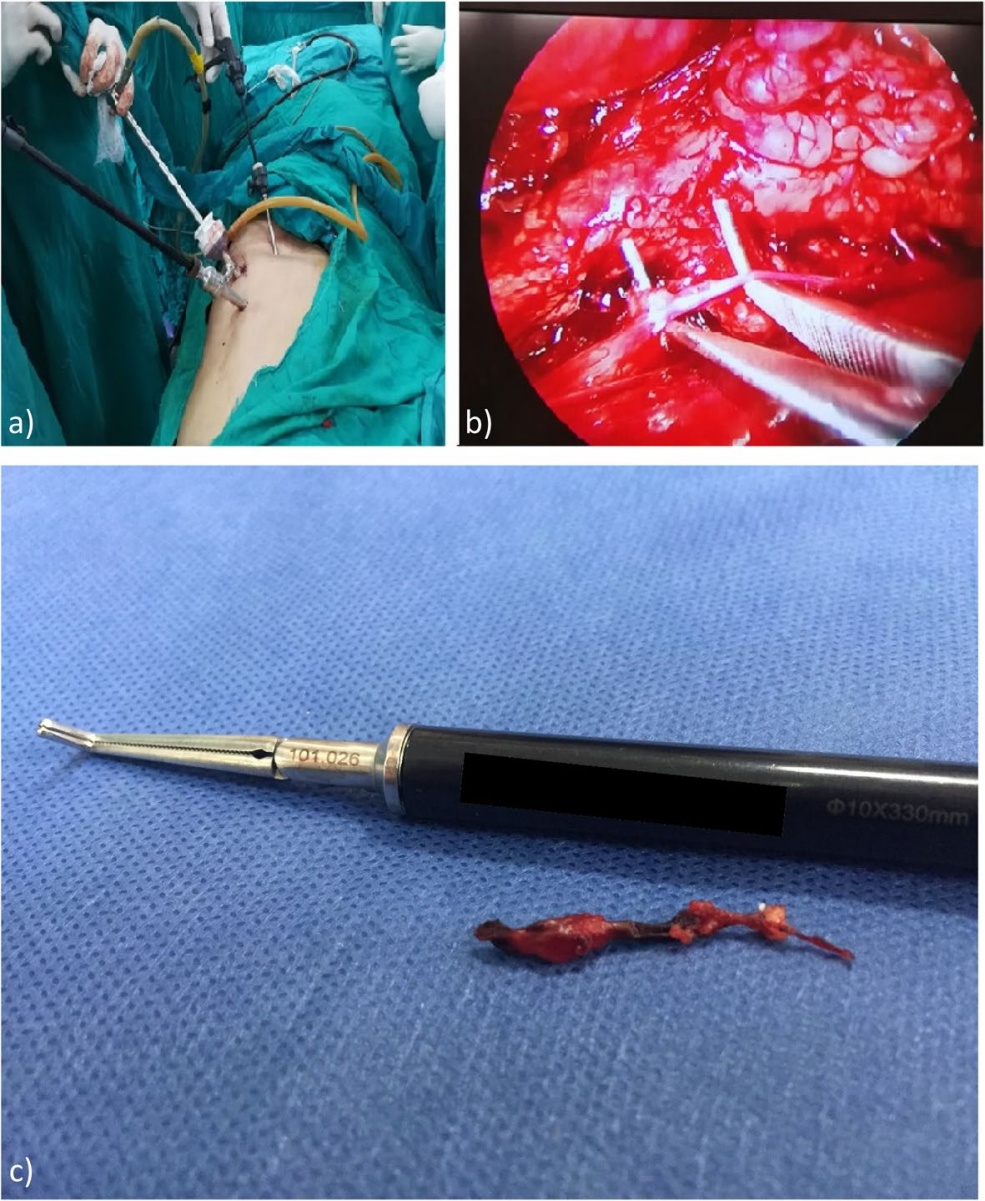
Laparoscopic Lumbar Sympathectomy: (**a**) Patient Position and trocar site (**b**) Laparoscopic view after sympathetic chain identification using a right angle clamp. **c** Resected Sympathetic Chain segment|

As Beglaibter N [4] described, a 10 mm incision is made midway between the iliac crest and the costal margin at the anterior axillary line. Blunt dissection using an artery clamp was commenced through the external and internal oblique muscles to reach the retroperitoneal space. A 10 mm trocar is then introduced into the space created and secured with two sutures to the fascia to avoid gas leakage. 30° scope is introduced into the trocar, and blunt dissection is started using the scope to create enough working space to place the subsequent trocars. Two additional 5 mm ports are inserted with direct vision into the retroperitoneal space along a line 2 to 3 cm anterior to the first trocar at the mid and posterior axillary lines. Two instruments are used for dissection, traction, clipping, and cutting, and sometimes for retraction of the psoas muscle (rolling out) for better exposure. The sympathetic chain is identified along the inner margin of the psoas muscle, and small communicating rami and blood vessels are divided with cautery, clips, and scissors. The sympathetic chain is then transected at the level of L2 and L4. The retroperitoneal space is deflated, the trocars are removed, the fascia at the large port site is sutured, and the skin incisions are closed. It is important to note that a single surgeon with advanced training in laparoscopic general surgery performed all RPLS. This helped to maintain consistency and ensure the standardization of the surgical protocol for patients who have undergone RPLS. For both techniques, once the sympathetic chain is identified, a right-angle clamp or a nerve hook removes at least two lumbar ganglia. It is then sent for histopathological confirmation Figure 2.

### Statistical analysis

Data computed from the COLS and RPLS group underwent a Shapiro–Wilk test for normality using the R statistical computing software version 4.4.1 [18]. Normally distributed data were reported in the form of mean and standard deviation, while data found with a skewed distribution from the normality test were reported in the form of median and interquartile range.

Data were analyzed using IBM SPSS software package version 20.0 [19]. Categorical data were collected in contingency tables and then analyzed regarding Chi-square (× 2), and a *p*-value less than 0.05 was considered significant. If any of the cells in the contingency table contained an event number less than 5, a Fisher’s-Exact test was employed for categorical data. An independent t-test (two-sample t-test) was used for continuous variables of normal distribution. R version 4.4.1 [18] were utilized for calculating the effect sizes; Hedge’s g was calculated for continuous variables, while the risk ratio was calculated for categorical variables. We used R version 4.4.1 [18] and G*Power 3.1 [20] software applications to conduct a post-hoc power analysis. A power value (1-β) greater than 0.8 constituted a sufficiently powered sample size to detect differences between groups.

## Results

The study included 24 patients divided into two groups: group I: RPLS (14 patients) and group II: COLS (10 patients). Table 1 illustrates the demographic and risk factors of the patients included.

**Table 1:** Baseline and Risk Factors

	**Group I** **(RPLS)** ***N* = 14**	**Group II** **(COLS)** ***N* = 10**	***P* value**
**Sex** (M/F)	12/2	8/2	0.21
Male	86%	80%	
**Age (years)**			
Mean	46.5 ± 10.3	48.3 ± 9.5	0.85
Range	35–55	30–60	
**Side affected** Lt/Rt	11/3	8/2	0.22
Percentage of Lt side	79%	80%	
**Rest pain**	14	10	0.32
VAS pain score	9.7 ± 0.89	9.4 ± 0.95	
Duration (months)	1.03 ± 0.37	1.82 ± 0.44	
**Trophic changes**			
Ulcer	1	1	0.41
Gangrene	-	1	
Duration (weeks)	4	3	
**Absent pulses**			
Femoral/popliteal/pedal	1 (7%)	-	
Popliteal/pedal	5 (36%)	4 (40%)	
Pedal	8 (57%)	6 (60%)	
**Capillary refill time**			
< 3 s	1	-	0.45
> 3 s	13	10	
**ABI**			
Mean	0.43 ± 0.08	0.38 ± 0.05	09
**TBI**			
Mean	0.38 + 0.06	0.35 + 0.05	0.41
**2OTcPO**_**2**_	25 + 9.9	30 + 3.3	0.11
**SF-36 Mean (± S.D)**	48 + 6.8	52 + 8.8	0.28
Smoking	7	8	0.73
Hypertension	6	4	0.23
Diabetes mellitus	5	6	0.32
Ischemic heart Disease	3	2	0.23
Dyslipidemia	6	5	0.32
Hypercoagulability	2	1	0.23
Malignancy	0	1	0.33
Vasculitis	1	0	0.24
Obesity	3	2	0.23

*N* number, *Lt/Rt* Left to right, *VAS* visual analogue scale, *RPLS* retroperitoneoscopic lumbar sympathectomy, *COLS* conventional open lumbar sympathectomy, *ABI* Anklebrachial index, *TcPo2* transcutaneous oxygen, *SF-36* 36-Item Short Form Heath Survey

Ischemic limb ulcers were present in 2 patients and limited gangrenous areas in 1 patient (distal foot gangrene). The risk factors in both groups are demonstrated in Table 1. All study population was on opiate analgesia in the form of Oxycontin tablets (40 – 80 mg/day). Postoperatively, although all patients were advised to discontinue opioids, 29% of group I (RPLS) and 20% of group II (COLS) still needed variable doses of opioid analgesia. All patients received Aspirin, statin, and naftidrofuryl before and after the intervention.

The operative results are summarized in Table 2. Technical success was achieved in all cases; proper identification of the lumbar chain was amenable in all the study populations. The mean operative time was significantly longer in the RPLS group, 86.4 ± 9.1 min, compared to 65.5 ± 8.6 min in the COLS group (*p*-value: 0.02). Operative time decreased with each subsequent case in the RPLS group; our analysis revealed a statistically significant reduction in the mean operative time for the first 5 cases and the other subsequent 9 cases in the RPLS group (*p*-value < 0.001).

**Table 2:** Intra-Operative Results

	Group I RPLS *N* = 14	Group II COLS *N* = 10	*P* value	Effect size [95% C.I]	Power (1- β)
**Operative time (min)**					
Mean + SD (range)	86.4 ± 9.1 (80–95)	65.5 ± 8.6 (60–75)	0.02*	Hedge’s g: 2.27 [1.22, 3.32]	0.99
Mean of first 5 cases	100.6 ± 11	-			
Mean of subsequent cases	62.2 ± 9	-			
**Operative Blood Loss (Ml)**					
Mean + SD	110 ± 12	420 ± 23	0.03*	Hedge’s g: 17.24 [12.12, 22.35]	1
Range	100–150	150–300			
**Conversion to Open**	1 (7.14%)				
**Intraoperative Complications**	2 (14.28%)	4 (40%)	0.03*	Risk Ratio: 2.80 [0.63, 12.43]	0.18
IVC injury	0	1			
Lumbar vessels injury	0	1			
Peritoneal tears	2	2			
Aortic injury	-	-			
Ureteric injury	-	-			
Nerve injury	-	-			

*N* number, *min* minutes, *IVC* inferior vena cava, * significant, *RPLS* retroperitoneoscopic lumbar sympathectomy, *COLS* conventional open lumbar sympathectomy

Intraoperative blood loss in COLS patients was significantly higher than in RPLS patients, 429 ± 23 and 110 ± 12 ml, respectively (*p*-value: 0.03). In the RPLS group, only one case (7.1%) was converted to open LS. Intraoperative complications occurred in 2 patients in the RPLS group and 4 cases in COLS group; 2 peritoneal tears with pneumoperitoneum in the RPLS group, and in COLS group, two peritoneal tears and 2 cases of bleeding (one from minor injury to IVC and a case from injured lumbar vessels). Injuries were repaired, and pneumoperitoneum was controlled by veress needle insertion in the hypochondrium.

The postoperative results at three months are illustrated in Tables 3 and 4. The mean hospital stay was significantly shorter in the RPLS group: 1 ± 0.5 and 2 ± 0.1 days in group I (RPLS) and group II (COLS), respectively (*p*-value: 0.02). The preoperative rest pain score decreased from 9.7 and 9.4 to 3.8 and 4.1 for group I (RPLS) and II (COLS), respectively. The analgesic effect lasted the whole study period in all subjects except one patient in the COLS group.

**Table 3:** Postoperative outcomes

		**Group I** RPLS ***N* = 14**	**Group II** COLS ***N* = 10**		***P* value**	**Effect size[95% C.I]**	**Power(1- β)**
**Length of Hospital stay** (days)		1 ± 0.5	2 ± 0.05		0.02*	Hedge’s g = 2.50 [1.41, 3.60]	**0.99**
**Need for opioid analgesia**		4	2				
**Changes in pain score:**							
Mean preoperative score		9.07 ± 0.6	8.9 ± 0.9	0.58		Hedge’s g for mean postoperative score between both groups = 0.37 [0.45, 1.18]	0.13
Mean postoperative score		3.35 ± 1.0	3.7 ± 0.8	0.39			
* P-value*		< 0.001*	< 0.001*				
Hedge’s g [95% C.I]		6.73 [4.77, 8.70]	5.85 [3.76, 7.93]				
Power (1- β)		**1**	**1**				
**Ulcer healing**			Yes			No	
**Need for amputation**			-			1	
**Capillary refill time (> 3 s)**							
Preoperative	13		10	0.16		Risk Ratio for postoperative capillary refill time > 3 s = 1.05 .30,0[3.69]	0.02
Postoperative	4		3	0.97			
**ABI**							
Mean preoperative score	0.43 ± 0.08		0.38 ± 0.05	0.09		Hedge’s g for mean postoperative score between both groups = 0.87 [0.02, 1.72]	0.52
Mean postoperative score	0.41 ± 0.07		0.36 ± 0.02	0.13			
* P-value*	0.38		0.12				
Hedge’s [95% C.I]	0.26[ -0.49, 1.00]		0.39, -0 [0.51.39]				
Power (1- β)	0.09		0.21				
**TBI**							
Mean preoperative score	0.38 + 0.06		0.35 + 0.5	0.41		Hedge’s g for mean postoperative score between both groups = 0.32 [-0.49, 1.14]	0.11
Mean postoperative score	0.55 + 0.02		0.54 + 0.04	0.32			
* P-value*	< 0.001*		< 0.001*				
Hedge’s [95% C.I]	3.69 [2.45, 4.93]		4.01 [2.45, 5.59]				
Power (1-β)	1		1				
**TcPO2 (mmHg)**							
Mean preoperative score	25 + 9.9		30 + 3.3	0.11		Hedge’s g for mean postoperative score between both groups = 0.69 [0.15, 1.53]	0.36
Mean postoperative score	45 + 6.6		50 + 7.5	0.10			
* P-value*	< 0.001*		< 0.001*				
Hedge’s [95% C.I]	2.31 [1.34, 3.28]		3.31 [1.92, 4.69]				
Power (1-β)	**0.99**		1				
**SF-36** Mean (± S.D)						Hedge’s g for mean postoperative score between both groups = 6.12 [4.16, 8.09]	**1**
Mean preoperative score	48 + 6.8		52 + 8.8	0.287			
Mean postoperative score	81 + 4.4		59 + 12	< 0.001*			
* P-value*	< 0.001*		0.521				
Hedge’s g [95% C.I]	5.59 [3.91, 7.28]		1.07 [0.13, 2.01]				
Power (1-β)	**1**		0.70				

*N* number, * significant, *RPLS* retroperitoneoscopic lumbar sympathectomy, *COLS* conventional open lumbar sympathectomy

**Table 4:** Postoperative Complications

	**Group I** RPLS ***N* = 14**	**Group II** COLS ***N* = 10**
Retroperitoneal Bleeding	-	-
Wound Pain	-	7
Wound Bleeding	-	1
Infection	-	2
Dehiscence	-	-
Incisional Hernia	-	2

*N* number, *RPLS* retroperitoneoscopic lumbar sympathectomy, *COLS* conventional open lumbar sympathectomy

As regards the two patients with foot ulcers, one patient in the RPLS group healed during the first postoperative month and continued to improve till the end of the study, while the other patient in the COLS group failed to recover and his presentation of a chronic toe ischemic ulcer persisted (punched-out ulcer with a necrotic base and no improvement in granulation tissue post-COLS). The patient with distal foot gangrene required a limited amputation, which was still healing at three months. The change in capillary refill time, ABI, TBI, and TcPO2 were not statistically significant between the two groups (*p*-value > 0.05). However, the difference in the quality-of-life score was statistically significant; the mean (+ SD) SF-36 score increased from 48 + 6.8 to 81 + 4.4 (*p*-value < 0.001) in group I (RPLS) compared to 52 + 8.8 to 59 + 1.2 (*p*-value: 0.52) in group II (COLS).

The incidence of postoperative wound pain was significantly higher in the COLS group, 70%, versus none in the RPLS group (*p*-value < 0.001). Two patients had superficial wound infection, and one had mild wound bleeding, controlled by conservative measures. Another two patients had an incisional hernia. Group I (RPLS) patients had no complications. None of the histopathological specimens were reported as anything apart from the sympathetic chain.

Results from our post-hoc power analysis revealed our study’s sample size was sufficiently powered (1- β greater than 0.8) for variables with statistically significant differences between RPLS and COLS groups. A notable exception was our calculated power of 0.18 in the comparison of post-operative complications between RPLS and COLS groups. An in-depth breakdown of the results of our effect size calculations and post-hoc power analyses is provided in Tables 2 and 3.

## Discussion

Although the gold standard treatment of critical limb-threatening ischemia (CLTI) is urgent revascularization, some patients have unreconstructable PAD for whom lumbar sympathectomy (LS) could still have a role in alleviating pain. The few randomized trials that compared LS to conservative treatment in CLTI cases failed to show beneficial effects on objective endpoints like amputation rate, mortality, or ABI. Still, it showed a beneficial impact on rest pain [21–26]. As revealed by Cochrane’s 2016 systematic review of clinical trial databases [27], no randomized trials comparing different lumbar sympathectomy techniques for CLTI exist. To the best of our knowledge, the present work illustrates the first double-armed comparison of the results of retroperitoneoscopic LS (RPLS) with conventional open LS (COLS) in non-reconstructable CLTI cases.

There was no statistically significant difference in the age, sex, risk factors, severity and duration of rest pain, trophic changes, absence of lower limb pulses, capillary refilling time, ankle-brachial index (ABI), toe-brachial index (TBI), TcPO2 and the 36-Item Short Form Health Survey (SF-36) in both groups of patients (*p*-value > 0.05). The operative time was significantly longer in the RPLS group (*p*-value: 0.02). Intraoperative blood loss, operative injuries, postoperative complications, and incidence of postoperative wound pain were substantially lower than in COLS patients (*p*-values: 0.03, 0.03, < 0.001, < 0.001, respectively). The differences in post-operative capillary refill time, ABI, TBI, and TcPO2 were not statistically significant between both groups (*p*-values: 0.97, 0.13, 0.32, 0.10, respectively). The mean hospital stay was significantly shorter in the RPLS group (*p*-value: 0.02). There was a statistically significant improvement in quality of life in the RPLS group compared to the COLS group (*p*-value: < 0.001).

The mean operative time was significantly longer in the RPLS group, 86.4 ± 9.1 min, compared to 65.5 ± 8.6 min in the COLS group (*p*-value = 0.02). Of note, the mean operative time in the first five cases of RPLS was 100.6 ± 11 min and decreased significantly to 62.2 ± 9 min in the last cases (*p*-value < 0.001), which underlines the importance of training and the rapid improvement in the learning curve. Sardinha et al., who have operated on 31 patients of RPLS, reported that the mean time of surgical procedure in the first 15 cases was 121 min and showed a significant reduction in surgical time in more recent cases to a mean of 87 min, reflecting an improvement in the learning curve [28]. It is important to note that a single surgeon with advanced training in laparoscopic general surgery performed all RPLS. This can explain the steady and rapid improvement in operative time.

Intraoperative adverse events were generally lower in the RPLS group, with significantly lower blood loss (*p*-value: 0.03) and no vascular injuries. The magnification and better visualization during the RPLS procedure could explain this. Loureiro et al., who assessed RPLS for foot hyperhidrosis, had no vascular injuries or injury to the ureter or genitofemoral nerve [29]. In our study, only one patient was converted from RPLS to OCOLS; this patient had a BMI of 37, which hindered proper trocar placement and decreased the working space. In the RPLS group, the procedure was completed laparoscopically despite two patients having a minor peritoneal tear. This can be because the primary laparoscopic surgeon had good experience with other extraperitoneal procedures, which allowed him to perform a verses needle to vent out the intraperitoneal space and, therefore, maintain a good working space.

Hospital stays, intra-operative bleeding, and postoperative wound pain were significantly lower in the RPLS group (*p*-values: 0.02, 0.03, < 0.001, respectively). Loureiro et al. reported a hospital stay between 1 and 3 days with a mean of 2.3 days, which agrees with our results [29]. Early postoperative complications occurred in the COLS group, with one patient having postoperative bleeding on day two that was controlled by conservative measures. Another two patients had mild abdominal wound infections, treated with antibiotics based on cultures and daily dressing. Incisional hernia developed in 2 patients of the COLS group during late follow-up. These data elaborate on the superiority of laparoscopic techniques in shortening the recovery period and lowering the rate of peri-operative complications.

Postoperative improvement in rest pain occurred in 23 patients, regardless of the technique adopted. The improvement persisted during the 3-month period of the study. Despite pain improvement, there was no amelioration in the presenting clinical signs: absent pulses, capillary refilling, ABI, TBI, or TcPO2. One of the patients with ulcers healed after surgery, and the patient with foot gangrene had a limited amputation. Similar to our findings, Lesiak et al. reported that LS didn’t affect the ABI [30]. Fazeli et al. also reported a 30% postoperative amputation rate; ischemic ulcers and gangrene were present in 20–23% at baseline [31]. Bozhurt et al. showed that LS led to improvement in symptoms in 52.3%, stable circulation in 27.8%, complications in 19.8% of the patients, and seven major and 36 minor amputations were performed [32]. A prospective study conducted by Ahmed et al. 2024 concluded that patients suffering from non-reconstructable PAD who underwent RPLS reported fewer wound complications and a decline in the length of hospitalization. However, a significant limitation of this study is the lack of a COLS group (control group) [14]. Similar findings were demonstrated by Nemes et al., which lacked a control group as well [33].

Of note, our study group included diabetics. Diabetics are a high-risk population for development of ischemic ulcers [34]. As our cohort of patients with CLTI included merely 2 patients with ischemic ulcers, we are unable to fully ascertain RPLS superiority over COLS in inducing the healing of ulcers. Generally, diabetics could have auto-sympathectomy as a late complication of diabetic neuropathy [35]. However, patients included in our study reported a short duration of diabetes; the incidence of diabetic neuropathy in patients with a recent onset of diabetes is between 7 and 15% [36]. Furthermore, there wasn’t a statistically significant difference in the incidence of diabetes between both groups (*p*-value: 0.33). Nevertheless, we recommend further studies regarding this issue to identify the population for whom the maximum benefit of LS will be achieved by excluding patients with severe neuropathy.

## Strengths and limitations

Among the strengths were being a comparative prospective study and using microcirculatory parameters to compare the results of the two techniques. On the other hand, the study's small number of the study population and the short-term follow-up period were the main limitations. Despite us not conducting an a priori power analysis, we calculated a post-hoc power analysis for each of our measured variables. Results from our retrospective power analysis demonstrated sufficient power to detect differences between both COLS and ROLS groups and therefore a low risk of falling into a type II error. However, our chosen sample size was not sufficiently powered to establish a significant difference between COLS and RPLS groups in terms of intra-operative complications. Whether the positive findings illustrated in the RPLS group would be evident beyond 3 months of the procedure remains unknown. Therefore, we recommend an exploration of the retroperitoneal approach’s longer-term follow-up outcomes in comparison with the open technique. The employment of a single-surgeon factor in RPLS procedures has helped maintain the consistency of surgical practices across all patients. We also maintained our internal validity by appropriately using allocation concealment to prevent selection bias by the surgeon. Despite this, we cannot fully ascertain our study’s external validity in other centers employing surgeons with different practices.

## Conclusion

Retropertoneoscopic lumbar sympathectomy (RPLS) is feasible, safe, and has the advantages of minimally invasive surgery: minimal blood loss, less intraoperative complications, shorter hospital stay, and less postoperative pain and complications. These compelling clinical findings empower vascular surgeons with the knowledge to confidently choose between retroperitoneal and open lumbar sympathectomy, ultimately benefiting CLTI patients facing this critical decision. However, the operative time in RPLS cases is longer than in the COLS; training on the procedure is recommended to improve the learning curve. Further randomized, controlled, and blinded clinical trials with larger sample sizes and longer follow-up periods are warranted to confirm our observations.

## Supplementary Information

Supplementary Material 1.

## Data Availability

No datasets were generated or analysed during the current study.
